# Phantom Epistasis in Genomic Selection: On the Predictive Ability of Epistatic Models

**DOI:** 10.1534/g3.120.401300

**Published:** 2020-07-23

**Authors:** Matías F. Schrauf, Johannes W. R. Martini, Henner Simianer, Gustavo de los Campos, Rodolfo Cantet, Jan Freudenthal, Arthur Korte, Sebastián Munilla

**Affiliations:** * Universidad de Buenos Aires, Facultad de Agronomía, Buenos Aires, Argentina; † International Maize and Wheat Improvement Center (CIMMYT), Texcoco, Mexico; ‡ Center for Integrated Breeding Research, Department of Animal Sciences, University of Göttingen, Germany; § Department of Epidemiology and Biostatistics, Michigan State University, East Lansing; ** Center for Computational and Theoretical Biology (CCTB), University of Würzburg, Germany; †† National Scientific and Technical Research Council (CONICET), Argentina

**Keywords:** Epistasis, Additive Effects, Genomics, Breeding, GenPred, Genomic Prediction, Shared data resources

## Abstract

Genomic selection uses whole-genome marker models to predict phenotypes or genetic values for complex traits. Some of these models fit interaction terms between markers, and are therefore called epistatic. The biological interpretation of the corresponding fitted effects is not straightforward and there is the threat of overinterpreting their functional meaning. Here we show that the predictive ability of epistatic models relative to additive models can change with the density of the marker panel. In more detail, we show that for publicly available Arabidopsis and rice datasets, an initial superiority of epistatic models over additive models, which can be observed at a lower marker density, vanishes when the number of markers increases. We relate these observations to earlier results reported in the context of association studies which showed that detecting statistical epistatic effects may not only be related to interactions in the underlying genetic architecture, but also to incomplete linkage disequilibrium at low marker density (“Phantom Epistasis”). Finally, we illustrate in a simulation study that due to phantom epistasis, epistatic models may also predict the genetic value of an underlying purely additive genetic architecture better than additive models, when the marker density is low. Our observations can encourage the use of genomic epistatic models with low density panels, and discourage their biological over-interpretation.

When reconciling the quantitative variation of complex traits with the Mendelian “scheme of inheritance”, Fisher (1918) presented a model where the genes contribute additively but also could interact in an epistatic manner. Nevertheless, he focused strongly on the additive component, regarding dominance, epistasis and environmental effects as “(nuisance) noise” (Price 1972). This started the tradition of mainly modeling additive effects, which is today’s standard in breeding programs (Crow 2010).

Naturally, the first approaches to predict genetic values with whole-genome marker panels were based on purely additive models (Meuwissen *et al.* 2001) and approaches including dominance and epistatic effects between markers have been subsequently proposed (Toro and Varona 2010; Su *et al.* 2012). While the latter seem to fit better to the molecular biological consensus that epistatic interactions are ubiquitous on a functional level (Mackay 2014), additive models continue to be predominant in genomics due to some advantages: a) additive models are easier to specify, b) dominant and epistatic variance components can be difficult to estimate with reasonable precision, c) most of the variance generated by genes with a dominant or an epistatic mode of action can be captured using an additive model, and d) for breeding purposes the prediction target we are most interested in is the additive component (Hill *et al.* 2008). This last argument (d) implicitly assumes that an additive model will more accurately predict the additive genetic value, relative to a non-additive model.

On the other hand, the non-additive models have been proposed with the expectation to a) increase the predictive ability of overall genetic values, b) allow the definition of mate allocation between candidates for selection, and c) exploit non-additive genetic variation through the definition of appropriate cross-breeds or purebred schemes (Varona *et al.* 2018). The latter assumes that these models actually capture non-additive effects. Early research demonstrated a predictive advantage of non-additive models (either parametric models including non-additive effects or non-parametric procedures such as kernel regressions or neural networks), but the results were often based on low-density genotype data (Crossa *et al.* 2010; de los Campos *et al.* 2010; Heslot *et al.* 2012). This low density goes along with low levels of linkage disequilibrium (LD) between markers and unobserved causal loci. In the context of association studies, incomplete LD has been shown to generate an apparent epistasis, which has been called “phantom” epistasis (Wood *et al.* 2014; de los Campos *et al.* 2019).

In this work we explore how the difference in predictive ability of epistatic models and additive models is related to the density of the markers used for predictions, and put our observations in the context of phantom epistasis. We begin by revisiting the case of phantom epistasis in association studies, and argue that a similar situation can occur in a predictive setting. We illustrate this with two examples of real data, an Arabidopsis and a rice data set, where the prediction accuracy is higher for epistatic models when using low density marker panels, but this advantage vanishes with increasing marker density. We then perform a simulation replicating the pattern observed for the real data. Since the simulated underlying genetic architecture is purely additive, this illustrates that epistatic models may also predict an additive genetic value better than an additive model, when the marker density is insufficient. We finish by discussing the consequences for the biological interpretation of genomic models.

## Theoretical background

While it is clear from molecular biology that the functional interaction of gene products is a key concept of biological systems, statistical models often generate good description of these systems when only using additive effects (Hill *et al.* 2008). This circumstance raises the question of whether statistically detected interaction effects between markers also reflect a particularly important functional interaction. After association studies detected many instances of epistasis between pairs of genetic variants (Hemani *et al.* 2014), Wood *et al.* (2014) proposed an alternative interpretation to the functional hypothesis that was being held. They showed that for most detected interacting pairs, a single third locus could explain all of this “apparent” epistasis. Following this, Zan *et al.* (2018) strengthened the case for a non-functional explanation of statistical epistasis, generated by high order linkage disequilibrium (LD) between the genotyped markers and unobserved functional polymorphisms. Finally, de los Campos *et al.* (2019) showed with a simple three loci model the specific conditions where the apparent epistatic effects between markers could arise from incomplete linkage disequilibrium with a single unobserved causal locus, even if the locus effect was fully additive. Because of the “illusive” nature of these apparent effects they coined the term phantom epistasis.

It is reasonable to suppose that an analogous situation to this phantom epistasis could also affect the predictive performance of additive and epistatic models for genomic prediction, despite its conceptual differences from association studies. Here the goal is to use the whole-genome markers for the prediction of genetic values or phenotypes, rather than for unraveling the genetic architecture. As such, the criterion of statistical significance is replaced by the one of predictive ability. In this context we deem an effect relevant when it’s inclusion in the model increases the model’s predictive ability.

Increasingly, genomic models are being specified not via explicit marker effects, but rather with Genomic Relationship Matrices (GRMs), which parametrize the covariance between genetic values. For additive models, the G matrix of VanRaden (2008) can be considered as the standard. Epistatic models can be specified with different GRMs; for example, in the “Extended G-BLUP” model (EG-BLUP), the GRMs is obtained as the Hadamard square of the additive GRM (Su *et al.* 2012; Jiang and Reif 2015; Martini *et al.* 2016). Also, the use of the Gaussian Kernel (de los Campos *et al.* 2010; Morota and Gianola 2014) results in an epistatic genomic model. Notice that the terminology of additive, dominant and epistatic is carried over to the genomic models. The classification of covariance matrices - classically defined in terms of pedigree-relationships (Henderson *et al.* 1984) - into additive and non-additive matrices has been extensively borrowed for the GRMs (Martini *et al.* 2016; Varona *et al.* 2018). This widespread use has one justification, among others, in the equivalence of genomic models which use GRMs to genomic models which refer to marker effects explicitly.

It is well known that, for a suitable choice of parameters, the additive G-BLUP is equivalent to a ridge regression on the markers (see Strandén and Christensen 2011). Because this ridge regression model has no interactions between marker effects, the equivalent G-BLUP is interpreted as an additive model. The first epistatic model considered in this work, the “Extended GBLUP”, has also been derived as a model equivalent to a ridge regression, this time on the pairwise marker interactions (see Jiang and Reif 2015; Martini *et al.* 2016). And while it is less known, a model with the Gaussian kernel can also be seen as the limit of a series of regressions with marker interactions of arbitrary order, where the effects of higher order have increasing penalty and thus are of progressively smaller magnitude and contribute less and less to the model prediction (thus ensuring convergence of the limit, see Cotter *et al.* 2011).

We used the equivalent regression models to partition the genotypic values associated to these GRMs into additive and non-additive components. Because variance explained by additive effects can also be partly explained by interactions and vice-versa (Huang and Mackay 2016), the additive variance has been given precedence to the epistatic variance, and lower order epistatic effects precedence to higher order effects. The detailed algorithm is given in the Materials and Methods section, together with explicit formulas for the GRMs entries and the terms in the corresponding equivalent ridge regressions.

## Materials and Methods

## Datasets

The first dataset consisted of the complete genomes of inbred lines of *Arabidopsis thaliana*, generated in the 1001 genomes project. This project provided 1,135 high-quality re-sequenced natural inbred lines representing the native Eurasian and North African range and the recently colonized North America populations (Alonso-Blanco *et al.* 2016). Trait measurements for subsets of these lines are available in the phenotype database ”Arapheno” (Seren *et al.* 2017). In this work we used the trait ”days to flowering at 10°”, measured in a growth chamber study as it is available for 1035 of the sequenced lines. This trait is relevant to the local adaptation of the Arabidopsis varieties in their extensive geographical distribution (Koornneef *et al.* 2004).

The second dataset was made available by the 3,000 Rice Genomes Project (Wang *et al.* 2018). It consists of 2018 genotyped varieties of Oryza sativa from the isozyme groups aus, japonica and indica, with 4 million high-density SNPs called from sequenced data against the Nipponbare reference Os-Nipponbare-Reference-IRGSP-1.0. Phenotype data for the rice varieties came from the International Rice Genebank Collection Information System (IRGCIS). We used the trait “thousand seeds weight (TSW)”, which at the time of the study was the quantitative trait phenotyped for the largest number of genotyped varieties. This trait is one of the main components of agronomical yield, and thus a relevant target in plant breeding programs (Peng *et al.* 2000).

### Genomic relationship matrices and prediction models

To explore the relation between apparent epistasis and marker density, we created 5 different panels with a varying number of markers ranging from $10^{2}$ to $10^{6}$ markers in the respective datasets. Thus, we fully explored the range of typical marker densities used to date in genomic studies. We used only single-nucleotide polymorphisms (SNPs) as markers in our analyses, and removed those with a minor allele frequency (MAF) below 1%. For all datasets, the markers were chosen to be uniformly spaced.

From these marker panels of different density, we calculated three genomic relationship matrices (GRMs), namely the additive G-BLUP GRM according to VanRaden (2008), the epistatic matrix from the EG-BLUP (Ober *et al.* 2015; Jiang and Reif 2015; Martini *et al.* 2016; Vitezica *et al.* 2017), and the Gaussian kernel GRM (de los Campos *et al.* 2010). We used these GRMs as covariance structures to build the following mixed linear models (MLMs):

y=Xβ+Zu+ε (1)

where *y* is the predicted trait, *X* is the design matrix for fixed effects and *β* are the coefficients. In turn, *Z* is the design matrix connecting observed phenotypes to accessions and *u* is the vector of genotypic values for the accessions, with variance proportional to the respective GRM considered. For the additive models, we used the now standard *G* matrix from VanRaden (2008). For the epistatic models we used the matrix from equation (8) in Martini *et al.* (2016) and the Gaussian Kernel (Morota and Gianola 2014), here called *H* and *K*, respectively. We note that while *H* is used together with *G* in the EG-BLUP, here we use it by itself in a model we call H-BLUP. This is done to illustrate properties of *G* and *H* separately.

We give below explicit formulas for the entries of the GRMs, up to a multiplicative constant:

$$G_{ij}\propto \sum_{k=1}^{p}\left(m_{ik}-{\bar{m}}_{\cdot k}\right)\left(m_{jk}-{\bar{m}}_{\cdot k}\right)$$ (2)

$$H_{ij}\propto {\left(\overset{p}{\underset{k=1}{\sum }}m_{ik}m_{jk}\right)}^{2}$$ (3)

$$K_{ij}\propto \text{exp}\left(-\frac{1}{2h}\overset{p}{\underset{k=1}{\sum }}{\left(m_{ik}-m_{jk}\right)}^{2}\right)$$ (4)

where the $m_{ik}=\left\{0,1\right\}$ are minor allele indicator variables for accession *i* and marker *k*. (Notice a more standard description maps the marker state to the set {0,1,2}. However, as these are inbred lines, we have no heterozygous loci.) In addition, *h* stands for the bandwidth parameter defined here by half the median Euclidean distance between genotypic profiles (de los Campos *et al.* 2010). All GRMs were scaled to have trace equal to *n*, where *n* is the number of genotyped accessions.

As mentioned above, mixed models with these GRMs are equivalent to ridge regression models on the molecular markers. The terms in the regressions equivalent to the G and H-BLUP can be found in Strandén and Christensen (2011) and Martini *et al.* (2016), respectively. We give expressions for the regressor variables ($X_{:,J}$) in each equivalent regression, as functions from the accession’s marker alleles:

- For *G* there is a term for each marker *k*,
  $$X_{i,k}=m_{ik}-{\bar{m}}_{\cdot k}$$ (5)

where ${\bar{m}}_{\cdot k}$ is the mean entry for marker *k* over all individuals. These terms generate a typical ridge regression (on centered markers).

- For *H* there is a term for each pair of markers *k* and *l* (possibly the same),
  $$X_{i,kl}=m_{ik}m_{il}$$ (6)

These terms build a homogeneous quadratic polynomial.

- For K there is a term for each d-tuple of markers $J=\left\{k_{1},\ldots ,k_{d}\right\}$,
  $$X_{i,J}=\frac{e^{-\frac{\left\|m_{i\cdot }\right\|}{2h}}}{\sqrt{h^{d}d!}}\overset{\left|J\right|}{\underset{q=1}{\prod }}m_{iJ_{q}}$$ (7)

These terms build an infinite order polynomial, where the terms of lower order have associated higher weights and thus are “cheaper” to use by the regularized regression model (Cotter *et al.* 2011).

Regarding the fixed effects of the models, we explored a variety of ways to model the population structure. Five sets of predictors were considered, varying on how and to what extent they describe the population structure. They consisted of one predictor set with just a constant “mean” predictor, two predictor sets based on a genomic clustering of the accessions into 3 and 9 groups (Murtagh 1985), and two more predictor sets based on the first and first five principal components. We use the notation *μ*, CL3, CL9, PC1 and PC5, to distinguish between the different variants of fixed effects included in the model.

### Variance components decomposition

To estimate the contribution of a regressor variable ($X_{:,J}$) to additive and non-additive variance component we regressed $X_{:,J}$ on an auxiliary model; one which only has the markers included in the sampled terms and interactions up to one order less than the term regressed ($Z_{d-1}$). This auxiliary model partitioned $X_{:,J}$ into a prediction of lower order interactions ($X_{d-1}$) and a residual (*ε*). The variance of the residual was considered the contribution of $X_{:,J}$ to the non-additive variance component of highest order, then $X_{d-1}$ took the role of $X_{:,J}$ and contributions to lower order components were calculated recursively. The general procedure is summarized with pseudocode in Algorithm 1. Finally, to estimate the proportion of contributions for the whole GRMs we sampled *J* over the markers indices for *G* and over marker pairs for *H*. For *K*, we sampled separately for tuples of length 1 to 6. After verifying that the contribution to the higher orders was negligible, we averaged the six samples weighted by the number of tuples of each length. For the three GRMs, a sampling with $10^{4}$ repetitions ensured stable, replicable estimation.

### Real trait analysis

To explore the possibility of an apparent epistasis phenomenon on flowering time in Arabidopsis and seed weight in rice, we used all genotyped and phenotyped accessions of each dataset and fitted genomic models with the GRMs as described above. We fitted 75 models resulting from the 5 marker panels of varying density, the 3 GRMs and the 5 sets of fixed effects considered. To assess the predictive ability of these models, they were fitted 100 times, once for each of the training sets resulting from a 10 times replicated 10-fold cross-validation scheme (Kohavi 1995), and we retained the predictions over the corresponding testing sets. We then calculated the predictive abilities as the correlation of predictions and observed phenotypic values over the testing sets. To assess the significance of differences in predictive ability between the models we used bootstrapped confidence intervals for the relevant contrasts following Hothorn *et al.* (2005) with one modification: we resampled testing sets as blocks, rather than predictions individually.

### Simulated trait analysis

With the observed trait we have no control of the underlying genetic architecture. To remedy that, we performed a simulation study, where we implemented the same analysis with fully additive genetic effects. For this purpose, 999 of the polymorphisms in the sequence data were chosen to take part as proxy causal loci. These causal positions were chosen to be not included in the panels but rather mid-position between every consecutive pair of SNPs in the 10^3^ marker panel. This was done in order to maximize the number of markers in incomplete linkage with the causal positions.

The effects of the causal positions were randomly sampled from identically and independent Gaussian distributions. The phenotype was simply the sum of the contributions from all causal positions plus an independent error noise, such that the trait heritability was 0.6. Notice that with this scheme we ensure that all the gene action is additive. By sampling new genetic effects (on the same loci) we simulated ten different traits. Subsequently, we proceeded to conduct the same analysis as with the observed traits described above. The causal loci were excluded for the formation of the GRMs.

### Software

The GRMs, as well as their variance component decomposition, where built with custom code in the Julia programming language (Bezanson *et al.* 2017), available upon request from the corresponding author. The remaining analyses were done in the R programming language (R Core Team 2020). In particular, the mixed models were fitted with the EMMREML package (Akdemir and Godfrey 2015).

### Data availability

The article analyses datasets in the public domain. The Arabidopsis dataset can be found in https://arapheno.1001genomes.org/. The Rice dataset can be found in https://doi.org/10.7910/DVN/HGRSJG. Derived data supporting the conclusions of this study are available from the corresponding author upon request, and can be replicated with the procedures present within the article, figures, and tables.

## Results

**Figure 1:**
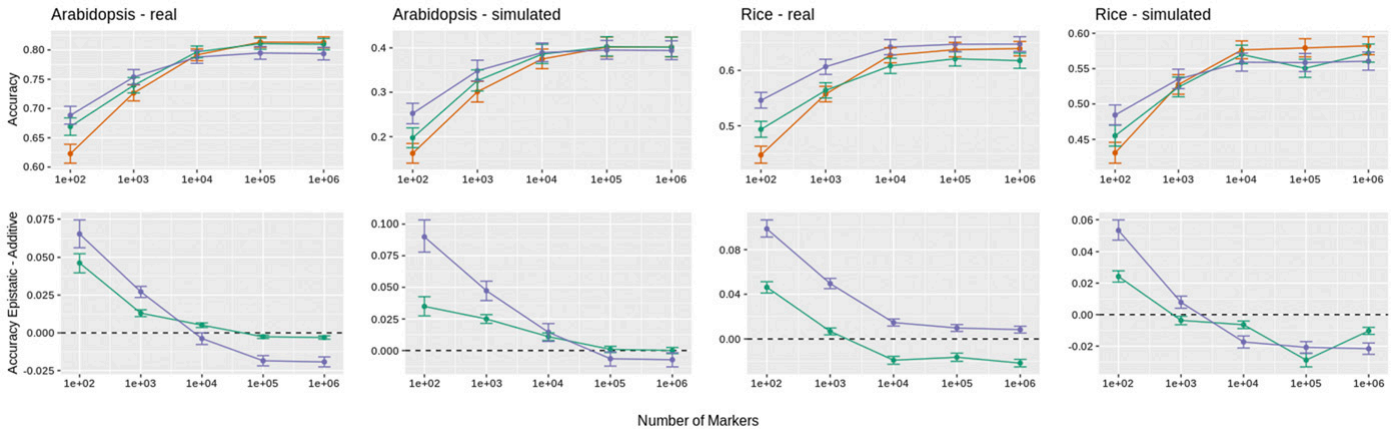
Predictive accuracy of genomic models for the real and the first simulated trait of *Arabidopsis* (left panels) and Rice (right panels). Upper panels: Mean predictive accuracy. Lower panel: Difference in predictive accuracy of epistatic models relative to the additive model. Error bars mark 99% bootstrap confidence intervals. GRMs: 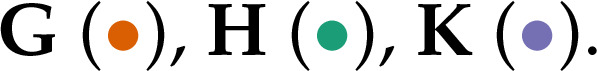

**Figure 2:**
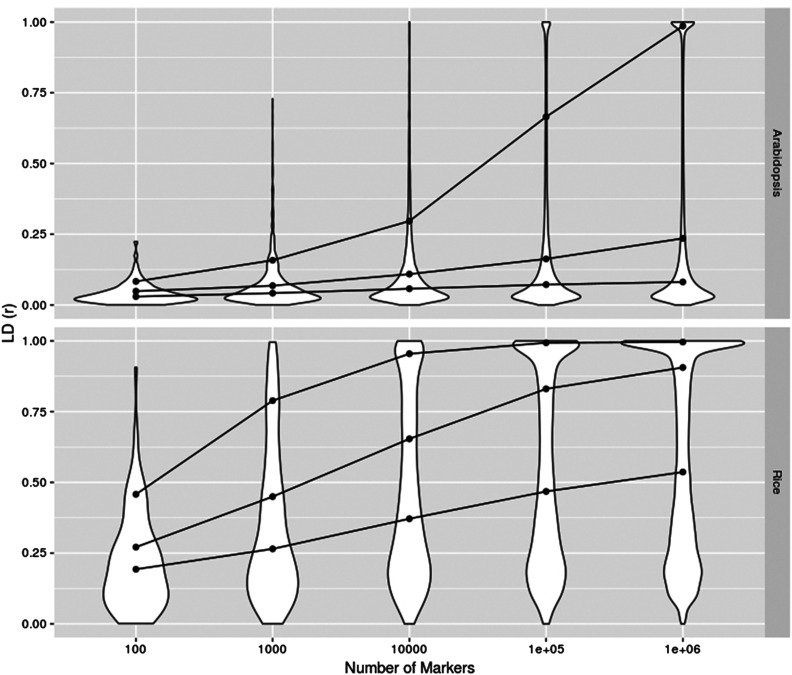
Linkage disequilibrium (*r*) between contiguous markers for panels with varying densities. Connected dots trace quantiles 0.9, 0.7 and 0.5 of the LD distributions.

Figure 1 illustrates the (average) prediction accuracies obtained with the trained models for the observed traits with additive and epistatic genomic models and their contrasts. Table 1 summarizes the relative accuracies obtained with the different marker panels for the observed traits and the averaged simulated traits. The summary is given as the difference between the G-BLUPs and the corresponding H and K-BLUPs. A supplementary picture is given in Table 2, with the percentage of cross-validation folds, cross-validation replicates and simulated traits where the epistatic models achieved higher average accuracy than the corresponding additive model. This shows the patterns are observed at all levels of aggregation. As expected, the panels with higher density had on average a higher LD between contiguous markers (Figure 2).

**Table 1:** Difference between the epistatic and additive models predictive accuracy (r) with 99% bootstrap confidence intervals

		$\left(r_{K}-r_{G}\right)$			$\left(r_{H}-r_{G}\right)$		
Traits	Panel	$\text{Δ}\bar{r}$	$CI_{.005}$	$CI_{.995}$	$\text{Δ}\bar{r}$	$CI_{.005}$	$CI_{.995}$
**Arabidopsis**							
real	$10^{2}$	0.066	0.057	0.074	0.046	0.040	0.053
	$10^{3}$	0.027	0.023	0.031	0.013	0.011	0.015
	$10^{4}$	−0.004	−0.008	0.000	0.005	0.004	0.007
	$10^{5}$	−0.019	−0.022	−0.015	−0.003	−0.004	−0.001
	$10^{6}$	−0.019	−0.023	−0.016	−0.003	−0.004	−0.002
simulated	$10^{2}$	0.061	0.057	0.065	0.030	0.027	0.032
	$10^{3}$	0.040	0.037	0.042	0.020	0.019	0.021
	$10^{4}$	0.004	0.002	0.006	0.006	0.005	0.007
	$10^{5}$	−0.009	−0.011	−0.007	−0.001	−0.002	0.000
	$10^{6}$	−0.011	−0.013	−0.009	−0.002	−0.003	−0.001
**Rice**							
real	$10^{2}$	0.099	0.091	0.106	0.046	0.041	0.051
	$10^{3}$	0.050	0.045	0.054	0.007	0.004	0.010
	$10^{4}$	0.015	0.011	0.018	−0.019	−0.023	−0.016
	$10^{5}$	0.010	0.007	0.013	−0.016	−0.020	−0.013
	$10^{6}$	0.008	0.005	0.011	−0.022	−0.025	−0.018
simulated	$10^{2}$	0.034	0.031	0.036	0.011	0.009	0.012
	$10^{3}$	0.013	0.012	0.014	0.000	−0.001	0.001
	$10^{4}$	−0.009	−0.010	−0.008	−0.005	−0.006	−0.004
	$10^{5}$	−0.013	−0.014	−0.012	−0.027	−0.028	−0.025
	$10^{6}$	−0.013	−0.014	−0.012	−0.007	−0.008	−0.006

**Table 2:** Percentage of cross-validation folds, cross-validation replicates and simulated traits where the epistatic model achieved higher predictive accuracy (*r*) than the additive model

		$\text{\%}\left[r_{K}>r_{G}\right]$			$\text{\%}\left[r_{H}>r_{G}\right]$		
Traits	Panel	folds	replicates	traits	folds	replicates	traits
**Arabidopsis**							
real	$10^{2}$	94	100	—	94	100	—
	$10^{3}$	98	100	—	95	100	—
	$10^{4}$	41	0	—	81	100	—
	$10^{5}$	4	0	—	33	0	—
	$10^{6}$	3	0	—	29	0	—
simulated	$10^{2}$	88	100	100	84	100	100
	$10^{3}$	89	100	100	92	100	100
	$10^{4}$	56	62	70	69	92	90
	$10^{5}$	33	14	10	48	45	40
	$10^{6}$	30	9	10	43	32	40
**Rice**							
real	$10^{2}$	100	100	—	98	100	—
	$10^{3}$	99	100	—	72	100	—
	$10^{4}$	86	100	—	5	0	—
	$10^{5}$	78	100	—	12	0	—
	$10^{6}$	76	100	—	4	0	—
simulated	$10^{2}$	92	100	100	72	86	90
	$10^{3}$	80	91	90	47	47	50
	$10^{4}$	25	2	0	26	1	0
	$10^{5}$	15	1	0	4	0	0
	$10^{6}$	15	1	0	17	0	0

The most obvious pattern is an improvement for all models in their predictive ability with the increase in marker density (Figure 1). This improvement is marginally decreasing, with mostly no practical difference shown between models using the $10^{5}$ and $10^{6}$ marker panels. We observe indications for prevalent “apparent epistasis“ due to an “epistatic gap”: an average improvement of predictive ability for epistatic models, when compared to the additive ones. This epistatic gap is present in the low density panels and vanishes or reverses with increasing number of markers. Furthermore, this pattern observed for the real traits is replicated on the simulated traits, which are fully additive at the functional level of the causal loci.

For the Arabidopsis data, we estimated the variance components associated with additive and epistatic effects of varying orders for the 10^3^ marker panel (Table 3). The G matrix of van Raden is exclusively additive (with respect to terms on the markers), while H from the Extended G-BLUP contributed both to components additive and of (additive x additive) epistasis. The Gaussian Kernel (K) fitted epistatic interactions of higher order, with contributions from orders higher than four being negligible. The contributions of the Gaussian Kernel depended on the bandwidth parameter (*h*), so we quantified the components of K for two values of *h*: the usual ”default”, equal to half the median euclidean distance (K(0.5)) and another the quarter of the median euclidean distance (K(0.25)) with only the first one being used in the fitted models.

**Table 3:** Percentage of variance components’ contribution to total genetic variance, for different GRMs, at the level of the markers. GRMs marked with (*) shown to illustrate dependence of K to bandwidth parameter (*h*) and are not used in the fitted models

Species	GRM	%$\sigma^{2}A$	%$\sigma^{2}AA$	%$\sigma^{2}AAA$	%$\sigma^{2}A^{4}$
Arabidopsis	G	100.0	0.0	0.0	0.0
	H	63.5	36.5	0.0	0.0
	K(0.5)	79.6	18.8	1.5	0.0
	K(0.25)*	63.2	31.7	4.8	0.3
Rice	H	100.0	0.0	0.0	0.0
	G	78.5	21.5	0.0	0.0
	K(0.5)	83.5	15.5	1.0	0.0
	K(0.25)*	70.8	25.9	3.2	0.2

Regarding the fixed effects, the advantage of epistatic models with low density panels was stronger for models which did not model population structure (*μ*), and decreased when modeling more population structure variance as fixed effects (CL9 and PC5 compared with CL3 and PC1, respectively). All showed a significant epistatic gap with the same qualitative pattern (Table 4).

**Table 4:** Difference between the epistatic and additive models predictive accuracy (*r*) for all sets of fixed effects

		$\left(r_{K}-r_{G}\right)$					$\left(r_{H}-r_{G}\right)$				
Traits	Panel	*μ*	PC1	PC5	CL3	CL9	*μ*	PC1	PC5	CL3	CL9
**Arabidopsis**											
real	$10^{2}$	0.127	0.119	0.061	0.066	0.051	0.096	0.095	0.046	0.046	0.037
	$10^{3}$	0.035	0.035	0.028	0.027	0.026	0.015	0.015	0.014	0.013	0.014
	$10^{4}$	0.003	0.003	−0.003	−0.004	−0.004	0.007	0.007	0.006	0.005	0.005
	$10^{5}$	−0.012	−0.013	−0.019	−0.019	−0.019	−0.001	−0.001	−0.002	−0.003	−0.003
	$10^{6}$	−0.013	−0.014	−0.019	−0.019	−0.020	−0.001	−0.001	−0.003	−0.003	−0.003
simulated	$10^{2}$	0.068	0.067	0.048	0.061	0.048	0.034	0.034	0.021	0.030	0.022
	$10^{3}$	0.039	0.039	0.039	0.040	0.040	0.020	0.020	0.019	0.020	0.020
	$10^{4}$	0.005	0.005	0.004	0.004	0.002	0.006	0.006	0.006	0.006	0.006
	$10^{5}$	−0.008	−0.009	−0.009	−0.009	−0.011	−0.001	−0.001	−0.001	−0.001	−0.001
	$10^{6}$	−0.010	−0.010	−0.010	−0.011	−0.013	−0.002	−0.002	−0.002	−0.002	−0.002
**Rice**											
real	$10^{2}$	0.097	0.096	0.091	0.099	0.099	0.046	0.043	0.043	0.046	0.046
	$10^{3}$	0.049	0.050	0.050	0.050	0.050	0.006	0.005	0.006	0.007	0.006
	$10^{4}$	0.014	0.014	0.015	0.015	0.015	−0.020	−0.020	−0.020	−0.019	−0.019
	$10^{5}$	0.009	0.009	0.009	0.010	0.010	−0.017	−0.017	−0.017	−0.016	−0.016
	$10^{6}$	0.008	0.008	0.008	0.008	0.009	−0.022	−0.022	−0.022	−0.022	−0.022
simulated	$10^{2}$	0.034	0.034	0.027	0.034	0.033	0.011	0.012	0.009	0.011	0.010
	$10^{3}$	0.013	0.013	0.013	0.013	0.013	0.000	0.001	0.002	0.000	0.000
	$10^{4}$	−0.009	−0.009	−0.009	−0.009	−0.009	−0.005	−0.005	−0.005	−0.005	−0.005
	$10^{5}$	−0.012	−0.013	−0.013	−0.013	−0.013	−0.027	−0.027	−0.027	−0.027	−0.026
	$10^{6}$	−0.013	−0.013	−0.013	−0.013	−0.013	−0.007	−0.007	−0.007	−0.007	−0.007

## Discussion

### Predictive ability and marker density

The research field of genomic selection is strongly focused on the accuracy of predictions, and one of the impacts that non-additive models can have is a potential increase in the predictive performance (*cf*. Varona *et al.* 2018). Here, in the analyses of real phenotypes we also observed such an advantage of epistatic models when using low density marker panels (Figure 1). However, this relative improvement compared to an additive model vanished with increasing marker density. Consequently, one should be cautious to interpret improvements in prediction accuracies as indicative of the existence of non-additive effects. An alternative explanation based on the fact that, due to incomplete LD, SNPs may not be fully capturing additive effects at all causal loci is also plausible. The degree of phantom epistasis is likely to decrease with increasing number of markers because higher marker density also improves the LD between SNPs and causal variants. An important support for this alternative interpretation is that the very same pattern of changes of predictive ability was observed when simulating traits with a fully additive genetic architecture.

One may also argue from the opposite direction that even if an epistatic model better agrees with the genetic architecture, the accuracy of the additive models could still increase by the addition of non-causal markers. In this sense, Dai *et al.* (2020) explored how an agreement between model and true genetic architecture improves polygenic prediction. In addition, Platt *et al.* (2010) showed that additive effects associated to non-causal markers can be effective at describing epistatic interactions in complex traits. Nevertheless, it is difficult to see how such a situation could explain the patterns observed here, as the epistatic model could similarly increase in predictive accuracy with additional markers. Also, there is no a-priori reason to expect that epistatic models with few (non-screened) markers accurately capture the causal interactions. Especially for the arabidopsis dataset, if we consider how little LD exists between contiguous markers in the lower density panels.

It is also unlikely that our observations are related to the choice of statistical models which we used for the predictions, since this pattern appears to be robust across all scenarios explored. This includes the different fixed effects modeled, but also the different epistatic models and the different replicates of causal effects used in the simulations. We observed evidence for apparent epistasis in all the ten traits simulated for either dataset. In particular, it is suggestive that the epistatic gap with low density panels was bigger when using the Gaussian Kernel, which has the potential to fit epistatic variance of higher order than the H-BLUP or the EG-BLUP. Nevertheless, when we estimated the contribution to total genetic variance of additive and epistatic components of varying order for the three GRMs used (Table 3) the higher order terms in K were found to be contributing in very small proportions to the total genetic variance. Different choices of bandwidths result in Ks with a different partition between additive and non-additive components, which could be exploited with multi-kernel models (de los Campos *et al.* 2010). It should be noted that the decomposition we used into “orthogonal” variance components shares with similar decompositions (*e.g.*, Álvarez-Castro and Carlborg 2007) the shortcoming that it ignores higher order LD present in genome wide marker panels (Vitezica *et al.* 2017); therefore, results need to be interpreted with caution.

### Implications for genomic selection

There are many examples in breeding literature where variance components or effects derived from whole-genome regression are overinterpreted in a functional way. In the last few years several reports have been published addressing this overinterpretation. For instance, Huang and Mackay (2016) pointed out that it may lead to wrong conclusions when the magnitude of the variance components are interpreted with respect to the genetic architecture. Moreover, it has been highlighted that when using regularized regressions (both penalized and Bayesian methods) instead of ordinary least square regression, the coding of the markers has an impact on effect estimates (Martini *et al.* 2016, 2019), which indicated that the mathematical presetting may have a relevant impact on the outcome of the estimates. Here we transferred the concept of phantom epistasis from the original development in the context of association studies (Wood *et al.* 2014; Zan *et al.* 2018; de los Campos *et al.* 2019) to genomic selection, and used it to provide an alternative explanation for patterns observed while comparing additive to non-additive genomic models in terms of predictive performance.

While more research is needed to explore the relevance of apparent epistasis in different populations and settings, and to incorporate dominance into the picture, the consideration of phantom epistasis can already have at least two consequences for genomic selection. First, considering the possibility of phantom epistasis allows us to identify scenarios where we would expect epistatic models to perform better in prediction than additive models. The improvement in accuracy is small, but very consistent (see Table 2) and attainable simply by model choice. While there has been work on the relative performance of genomic models, the most solid results have focused on factors which are unobservable, or outside the control of the researchers and breeders (like effective population size and genetic architecture; Daetwyler *et al.* 2010). On the other hand, the choice of the genomic model to be used is under the control of those actors (Daetwyler *et al.* 2012). The consideration of apparent epistasis, in this respect, encourages the use of epistatic models for genomic breeding value prediction, particularly when working in structured populations with low density panels.

Second, in the context of breeding, the prediction of genotypic values is often focused on the additive component (called breeding value). This has been an obstacle for the applied use of non-additive models in many breeding plans (Varona *et al.* 2018), as the potential increase in accuracy is usually thought to be based on capturing non-additive variance components at the level of the causal loci (Crow 2010). Instead, the existence of apparent epistasis suggests that the accuracy can increase by capturing more additive variance (of causal loci) associated with non-additive apparent effects (on markers). This point can be seen quite clearly on the simulated traits, where the genotypic values equal the breeding values by virtue of the genetic architecture being fully additive.

## Conclusion

Phantom epistasis under certain conditions is a plausible explanation for the better performance of non-additive models even when non-additive variance on the level of causal loci is expected to be low. In this work we observed such better performance when predicting flowering time in *Arabidopsis thaliana* and seed weight in rice, while using low density markers. That these differences in performance vanished when using more markers was suggestive of phantom epistasis. We strengthen this claim by obtaining remarkably similar patterns with simulated traits with a fully additive genetic architecture. The results of this work show that the marker interactions in these genomic models can capture not only the epistatic, but also the additive component of unobserved causal factors.

**Algorithm 1** Calculate contribution to variance components of varying orders by regression variable $x_{:,J}$.

**Input:**

*M*: marker incidence matrix of size n×p

*J*: a tuple with the indices of the markers involved in the regression variable

*φ*: function from M[:,J] to regression variable, specifically one of (5), (6) or (7)

**Output:**

result: A *d*-length vector, with contribution to $\sigma_{A^{i}}^{2}$ in position *i*, for i=1,2,…,d

Auxiliary functions:

**Poly**(*X*,*d*): a matrix whose columns consists of all monomials of degree *d* and lower of *X*’s columns

**Ols**(*X*,*y*): predictions from regression of matrix *X* on vector *y*

1: **procedure** contributionvarcomp(M,J,φ)
2: d←Length(J)
3: result←Vector(length=d)
4: $X_{d}\leftarrow \varphi (M[:,J])$
5: n←d
6: **while**n>0**do**
7: $Z_{n-1}\leftarrow Poly(M[:,J],d-1)$
8: $X_{n-1}\leftarrow Ols\left(Z_{n-1},X_{n}\right)$
9: $\varepsilon_{n}\leftarrow X_{n}-X_{n-1}$
10: $result[n]\leftarrow Var\left(\varepsilon_{n}\right)$
11: $X_{n}\leftarrow X_{n-1}$
12: n←n−1
13: **return**result
